# Association of Frailty based on self-reported physical function with directly measured kidney function and mortality

**DOI:** 10.1186/s12882-015-0202-6

**Published:** 2015-12-09

**Authors:** Cynthia Delgado, Barbara A. Grimes, David V. Glidden, Michael Shlipak, Mark J. Sarnak, Kirsten L. Johansen

**Affiliations:** Nephrology Section, San Francisco VA Medical Center, San Francisco, CA USA; Division of Nephrology, University of California, 521 Parnassus Ave, Box 0532, San Francisco, CA 94143 USA; Department of Epidemiology and Biostatistics, University of California, San Francisco, CA USA; Department of Medicine, University of California, San Francisco, CA USA; Division of Nephrology, Tufts Medical Center, Boston, MA USA

**Keywords:** Self-report frailty, Kidney function and mortality

## Abstract

**Background:**

Use of serum creatinine to estimate GFR may lead to underestimation of the association between self-reported frailty and kidney function. Our objectives were to evaluate the association of measured GFR (mGFR) with self-reported frailty among patients with CKD and to determine whether self-reported frailty was associated with death after adjusting for mGFR.

**Methods:**

Participants in the Modification of Diet in Renal Disease study (1989–1993) had GFR measured using iothalamate clearance (mGFR), and GFR was estimated based on the CKD-EPI creatinine (eGFRcr) and cystatin C (eGFRcys) equations. We defined self-reported frailty as three or more of: exhaustion, poor physical function, low physical activity, and low body weight. Death was ascertained through 2007 using the National Death Index and the United States Renal Data System.

**Results:**

Eight hundred twelve MDRD participants (97 %) had complete data on self-reported frailty (16 % prevalence, *N* = 130) and mGFR (mean (SD) 33.1 ± 11.7 ml/min/1.73 m^2^). Higher GFR was associated with lower odds of self-reported frailty based on mGFR, (OR 0.71, 95 % CI 0.60–0.86 per 10 ml/min/1.73 m^2^), eGFRcr (OR 0.80, 95 % CI 0.67–0.94 per 10 ml/min/1.73 m^2^), and eGFRcys (OR 0.75, 95 % CI 0.62–0.90 per 10 ml/min/1.73 m^2^). Median follow-up was 17 (IQR 11–18) years, with 371 deaths. Self-reported frailty was associated with a higher risk of death (HR 1.71, 95 % CI 1.26–2.30), which was attenuated to a similar degree when mGFR (HR 1.48, 95 % CI 1.08–2.00), eGFRcr (HR 1.57, 95 % CI 1.15–2.10), or eGFRcys (HR 1.51, 95 % CI 1.10–2.10) was included as an indicator of kidney function.

**Conclusions:**

We found an inverse association between kidney function and self-reported frailty that was similar for mGFR, eGFR and eGFRcys. In this relatively healthy cohort of clinical trial participants with CKD, using serum creatinine to estimate GFR did not substantially alter the association of GFR with self-reported frailty or of self-reported frailty with death.

## Background

Frailty is more prevalent among patients with CKD than among individuals with normal kidney function [1–4], and frailty is also highly prevalent among non-elderly patients with ESRD [3, 4]. The association between frailty and adverse clinical outcomes is established in the ESRD population. Most studies of frailty and CKD have been among patients with ESRD, in community cohorts not specifically enriched for CKD [2, 5], or in CKD cohorts that employ estimates of kidney function limiting the ability to ascertain the strength of association between directly measured kidney function and frailty, independent of comorbidity and other factors.

The use of serum creatinine to estimate kidney function could also complicate the problem of assessing the association of kidney function with frailty. Because muscle wasting associated with frailty, there is a potential for bias using creatinine based estimates of glomerular filtration rate (eGFR). To our knowledge there have been no evaluations of the relationship of frailty with kidney disease using direct measurement of GFR. The Modification of Diet in Renal Disease (MDRD) study affords a unique opportunity to study frailty by self-report among a cohort of healthier patients who have stage 3 to 5 CKD not on dialysis. Within this cohort there is a wide range of directly-measured GFR to assess the association of kidney function with frailty. The purpose of this study was twofold; first to examine the association of kidney function with self-reported frailty, using a direct measure of kidney function and estimated GFR as a comparison, and second, to determine whether self-reported frailty was associated with death in this cohort with few comorbidities.

## Methods

### Study participants

The MDRD Study was a multicenter cooperative clinical trial designed to determine whether restriction of dietary protein and a low target blood pressure (mean arterial pressure <92 mmHg vs. usual target blood pressure (<107 mmHg)) reduced the rate of progression of CKD, irrespective of the nature of the primary underlying process. Patients with diabetes requiring insulin were excluded [6]. At baseline, the severity of kidney disease was assessed by measurement of GFR using iothalamate. Data on physical function and exercise were collected by questionnaires. Data from ancillary studies of this cohort that evaluated C-reactive protein (CRP) as a predictor of cardiovascular outcomes were included in this analysis [7]. Mortality outcomes were acquired via direct data collection within the MDRD study and through linkage with the National Death Index and the United States Renal Data System (USRDS) through December 12, 2007. Participants from MDRD study A and B were combined for the purpose of this analysis. Only participants with complete measures of physical functioning and linkage information were included in analyses (*n* = 812; 97 %). Informed consent was obtained from all participants as part of the original study. The Committee on Human Research at the University of California - San Francisco and the Research and Development Committee of the San Francisco Veterans Affairs Medical Center deemed this study exempt.

### Self-report frailty definition

Our frailty definition was an adaptation of the Fried Frailty Index that substituted patients’ self-report of physical function for the direct measures of physical performance (gait speed and grip strength) that are part of the original definition. This approach is similar to that originally developed by Woods et al. [8] and subsequently applied among patients with end-stage renal disease [9] and is (henceforth referred to as “self-reported frailty”) (Appendix). For the exhaustion criterion, participants responded to a symptom questionnaire that asked “In the past month how often have you felt lack of pep and energy? Tiring easily, weakness?” Patients were given one point in the exhaustion domain if their response to both questions corresponded to a moderate amount of time or more. The physical function domain was ascertained using the MDRD quality of wellbeing measure, which assessed participants’ ability to complete activities of daily living (ADLs) similar to the Rand SF-36 physical function scale. Individuals who scored in the lowest quartile based on normative data were allocated two points in the physical function domain of self-reported frailty. Physical activity was assessed using the MDRD Leisure Time Physical Activity Questionnaire, which measured the number of times per week each individual performed walking or other moderate and vigorous activities during leisure time. After converting physical activity into total kilocalories (kcal) of activity per week, individuals in the lowest quintile based on normative data [10] were allocated 1 point in the physical activity domain of self-reported frailty. The baseline assessment of standard body weight was used as a surrogate for weight loss. Individuals who were less than 95 % of standard body weight for sex and height were allocated 1 point in the weight loss/underweight domain of self-reported frailty. Self-reported frailty was defined by a score of ≥3 points, and patients scoring 1 or 2 points were considered intermediate frail by self-report from a total of 5 possible points [10].

### Measures of kidney function

Assessment of kidney function using clearance of ^125^I-iothalamate was completed twice during the baseline period for all participants. We used the average of the 2 baseline ^125^I-iothalamate clearance measures in our analyses (averaged measured GFR; mGFR). We modeled GFR as a continuous variable and as categories (≥45 ml/min/1.73 m^2^, 30 to 44 ml/min/1.73 m^2^, ≤ 29 ml/min/1.73 m^2^). To compare the association of the direct measurement of kidney function with self-reported frailty to the association of a creatinine-based estimate (eGFRcr) and cystatin C-based estimate (eGFRcys) of GFR with self-reported frailty, we used the Chronic Kidney Disease Epidemiology Collaboration [11] (CKD-EPI) formulas [12] using the same modeling strategies. We chose to use the CKD-EPI equation rather than the MDRD equation to evaluate the association between creatinine-based eGFR and self-reported frailty to avoid overestimating the similarity between mGFR and eGFRcr in the derivation cohort for the MDRD equation.

### Statistical analysis

Characteristics of participants who were frail by self-report, intermediate frail by self-report and non-frail were compared by Wilcoxon rank sum for continuous variables and chi square for categorical variables. We treated self-reported frailty as a dichotomous variable in the analyses comparing the different methods of measuring GFR with self-reported frailty, similar to previous studies in CKD and ESRD [2, 4]. In our survival analysis, self-reported frailty was treated as a three-level categorical variable (0 = not frail; 1–2 = intermediate frail, and 3 = frail by self-report) as Fried and others have done [2, 10, 13]. We used logistic regression modeling to estimate the association of mGFR, eGFRcr, and eGFRcys with self-reported frailty. With the exception of albumin and age, covariates included in the model were treated as continuous variables. Covariates included age (≤40 years, 41–59 years, ≥ 60 years), sex, quartiles of serum albumin concentration (≤3.84 mg/dL, 3.85–4.01 mg/dL, 4.02–4.24 mg/dL, ≥4.25 mg/dL), proteinuria, race, BMI, and log transformed CRP, protein intake and blood pressure group assignment. We tested for interactions between age greater than 60 and GFR and for interactions between sex and GFR in the adjusted models. A sensitivity analysis was conducted to determine whether associations were independent of study group assignment for blood pressure and protein. In addition, to examine the robustness of our findings, we examined the potential for non-linearity of continuous GFR predictors by including quadratic terms in the models and by examining the cubic splines. We tested whether the association of GFR with self-reported frailty differed according to method of GFR assessment by comparing the c statistics corresponding to areas under the ROC curves in adjusted models. Cox proportional hazards models were used to assess the association of frailty with death; mGFR, eGFRcr and eGFRcys were included as covariates in separate models. A *p*-value of less than 0.05 was considered statistically significant. Analyses were performed using SAS version 9.4 (SAS Institute Inc., Cary NC).

## Results

### Participant characteristics

Of the 840 MDRD participants, 812 had complete data for the assessment of self-reported frailty and were included in our analyses. The majority was male (60.5 %), the median age was 52 (interquartile range [IQR] 42–61), and a history of hypertension was common (84 %) (Table 1). As expected based on the MDRD inclusion criteria, very few participants had a history of diabetes (5 %). Approximately 21 % of participants had an mGFR greater than or equal to 45 ml/min/1.73 m^2^, 35 % had an mGFR between 30–44 ml/min/1.73 m^2^ and 44 % had an mGFR less than or equal to 29 ml/min/1.73 m^2^.

**Table 1:** Participant characteristics

Characteristic	Not frail (*N* = 252)	Intermediate frail (*N* = 430)	Frail (*N* = 130)	Total (*N* = 812)	*P*-Value
Sex, *n* (%) male	178 (70.6 %)	250 (58.1 %)	63 (48.5 %)	491 (60.5 %)	<0.001
Age, years	52 (43.5, 62)	50 (40,60)	56 (47,62)	52 (42,61)	<0.001
BMI, kg/m^2^	28 (3.9)	26.3 (4.6)	27.5 (5)	27 (4.5)	<0.001
Systolic blood pressure, mmHG	133 ± 14.7	133 ± 17.9	134 ± 16.7	133 ± 16.7	0.60
Race					
Caucasian	224 (89 %)	365 (84.9 %)	100 (76.9 %)	689 (85 %)	0.03
African American	15 (6.0 %)	37 (8.6 %)	12 (9.2 %)	64 (8 %)	
Other	13 (5.0 %)	28 (6.5 %)	18 (13.9 %)	59 (7 %)	
Renal parameters					
Baseline eGFRcys (ml/min/1.73 m^2^)	33.4 (12.8)	31.5 (12.6)	28 (11.3)	31.5 (12.6)	<0.001
Baseline eGFRcr (ml/min/1.73 m^2^)	34.4 (12.6)	32.9 (12.7)	30.2 (11)	32.9 (12.5)	0.007
Baseline mGFR (ml/min/1.73 m^2^)	35.1 (11.7)	32.8 (11.9)	30.0 (10.2)	33.1 (11.7)	<0.001
mGFR ≤29 ml/min/1.73 m^2^	86 (34.1 %)	198 (46.0 %)	75 (57.7 %)	359 (44.2 %)	<0.001
mGFR 30–44 ml/min/1.73 m^2^	100 (39.7 %)	141 (32.8 %)	42 (32.3 %)	283 (34.9 %)	
mGFR ≥45 ml/min/1.73 m^2^	66 (26.2 %)	91 (21.2 %)	13 (10.0 %)	170 (20.9 %)	
Serum creatinine, mg/dl	2.29 (0.89)	2.37 (0.94)	2.35 (0.82)	2.3 (0.90)	0.36
Serum cystatin C, mg/L, N = 797	2.11 ± 0.66	2.22 ± 0.67	2.36 ± 0.67	2.21 ± 0.67	0.001
Proteinuria, mg/L, N = 786	1.09 ± 1.75	1.1 ± 1.58	1.16 ± 1.91	1.11 ± 1.69	0.87
Additional laboratory parameters					
C-reactive protein^, mg/dL	0.24 (0.11,0.47)	0.20 (0.10,0.61)	0.31 (0.13, 0.71)	0.23 (0.11, 0.6)	0.07
Albumin, g/dL	4.03 (0.30)	4.02 (0.35)	4.0 (0.31)	4.02 (0.33)	0.77
Hematocrit, g/dl	40 (4.5)	38.6 (5.2)	38.1 (4.4)	38.8 (4.92)	0.004
Comorbid conditions					
Diabetes mellitus	12 (4.8 %)	18 (4.2 %)	12 (9.2 %)	42 (5.2 %)	0.07
Coronary artery disease	22 (8.7 %)	35 (8.1 %)	17 (13.1 %)	74 (9.1 %)	0.22
Hypertension	214 (84.9 %)	355 (82.6 %)	112 (86.2 %)	681 (84 %)	0.53
Etiology of renal disease					
Polycystic kidney disease	53 (21 %)	99 (23 %)	21 (16.2 %)	173 (21.3 %)	0.03
Glomerular disease	119 (47 %)	169 (39.3 %)	64 (49.2)	352 (43.3)	
Other	80 (32 %)	162 (37.7 %)	45 (34.6 %)	287 (35.3 %)	

*Abbreviations*: *BMI* Body mass index, Median (25th, 75th percentile) reported for Age. Footnote: ^For measurement of C-Reactive Protein, *N* = 781

### Prevalence of frailty

**Fig. 1:**
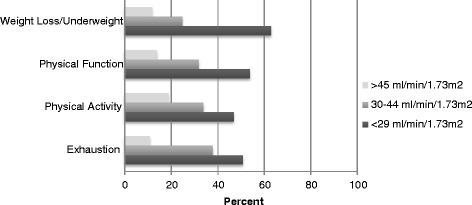
Prevalence of Frailty Components by mGFR category

The most common frailty components were low physical activity (47 %) and poor physical function (23 %), whereas a smaller proportion of individuals met the criteria for underweight/weight loss (17 %) and exhaustion (13 %). There was a graded association of the prevalence of the components of self-reported frailty with GFR category such that individuals in the lowest GFR category had a higher prevalence of each of the components of self-reported frailty (Fig. 1). Sixteen percent of patients were frail by self-report, 53 % were intermediate frail by self-report, and 31 % were not frail (Table 1). Self-reported frail individuals were slightly older than non-frail individuals. Self-reported frail patients were less likely to be male and less likely to be Caucasian. There were no statistically significant differences in the prevalence of hypertension or diabetes among self-reported frail and non-frail participants.

**Fig. 2:**
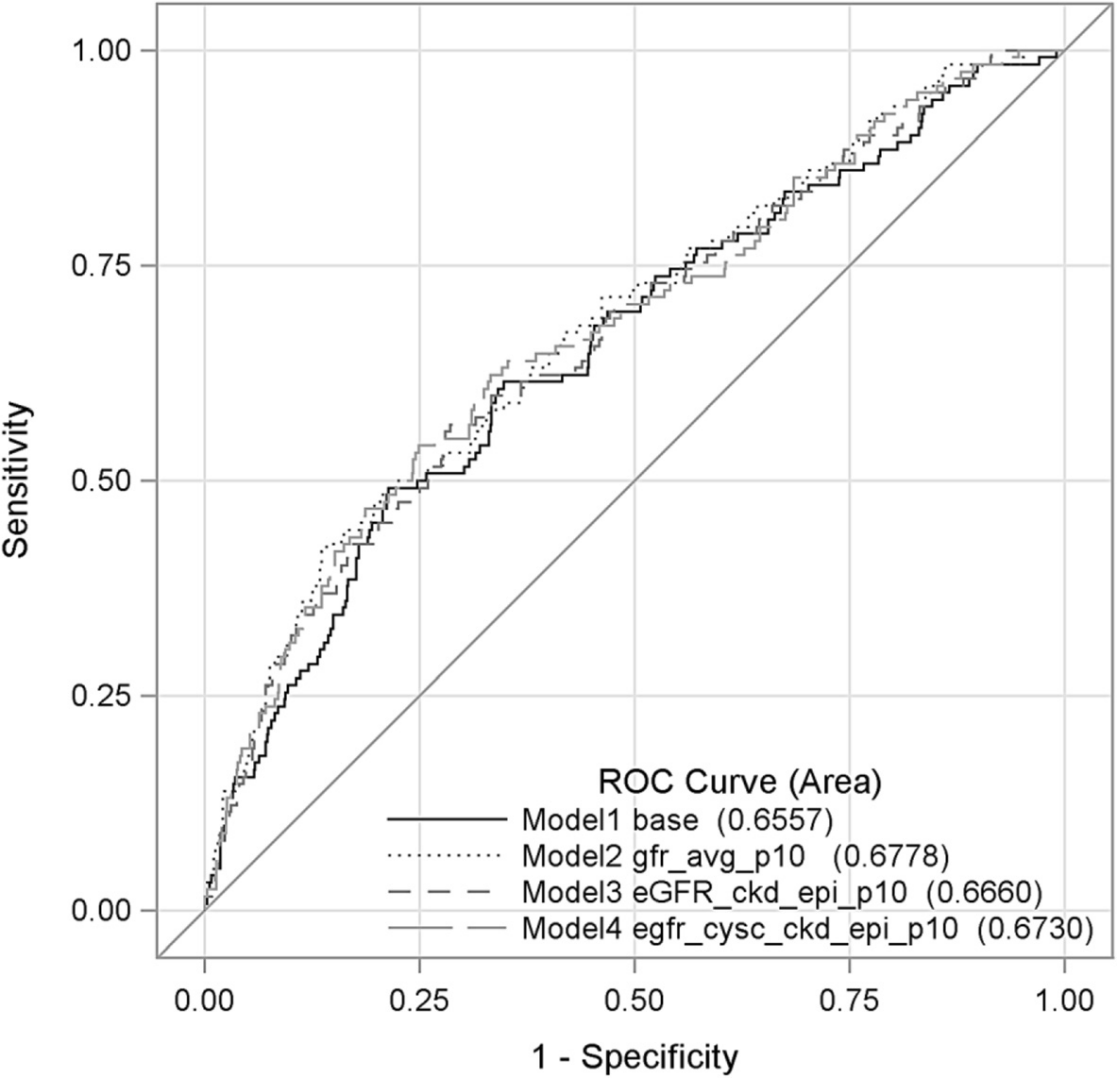
Area under the ROC curves. Legend: Model 1 (Base). Frailty, age, sex, BMI, race, proteinuria, serum albumin. Model 2: Base + mGFR, Model 3: Base + eGFRcr, Model 4: Base + eGFRcys

### Association of mGFR with self-reported frailty

In univariate analysis, higher mGFR was associated with lower odds of self-reported frailty (Table 2). C-reactive protein was not associated with frailty (OR 1.07, 95 % CI 0.95–1.2 per one log unit, data not shown) and was therefore not included in our final models. After adjusting for covariates, the association of mGFR with self-reported frailty remained statistically significant, with a similar point estimate as in the univariate analysis (OR 0.71 per 10 ml/min/1.73 m^2^, 95 % CI 0.60–0.86) (Table 2). When mGFR was considered in clinically relevant categories, in univariate analysis, individuals with mGFR 30–44 ml/min/1.73 m^2^ were more likely to be frail by self-report compared to mGFR ≥45 ml/min/1.73 m^2^ (OR 2.10 95 % CI 1.09–3.60). In fully adjusted modelling, this association was attenuated. **I**ndividuals with mGFR ≤29 ml/min/1.73 m^2^ were more likely to be frail by self-report than those with mGFR ≥ 45 ml/min/1.73 m^2^ (OR 3.40 95 % CI 1.77–6.50) in multivariable analysis.

**Table 2:** Cross-sectional association of GFR with self-reported frailty in the MDRD trial, using alternative measures of GFR

	mGFR		eGFRcr		eGFRcys*	
	Odds Ratio (95 % CI)		Odds Ratio (95 % CI)		Odds Ratio (95 % CI)	
	Univariate	Multivariable	Univariate	Multivariable	Univariate	Multivariable
GFR, per 10 ml/min/1.73 m2	0.75 (0.63–0.89)	0.71 (0.60–0.86)	0.81 (0.68–0.94)	0.80 (0.67–0.94)	0.74 (0.62–0.88)	0.75 (0.62–0.90)
GFR in categories						
GFR ≥45 (reference)	1.00	–	1.00	–	1.00	–
GFR 30–44	2.10 (1.09–4.00)	1.83 (0.93–3.60)	2.10 (1.04–4.20)	1.77 (0.87–3.60)	1.71 (0.82–3.60)	1.50 (0.70–3.20)
GFR ≤29	3.20 (1.71–5.90)	3.40 (1.77–6.50)	3.30 (1.72–6.50)	3.20 (1.61–6.40)	2.50 (1.25–5.00)	2.30 (1.11–4.70)

Abbreviations: mGFR, measured glomerular filtration rate by Iothalamate; eGFRcr, creatinine estimated glomerular filtration rate; eGFRcys by CKD-EPI equation,* *N* = 797. All multivariate models are adjusted for urine protein, sex, age, race, body mass index and albumin quartiles

### Association of estimated GFR (eGFRcr and eGFRcys) with self-reported frailty

Associations of eGFRcr with self-reported frailty were similar to those observed with mGFR. Higher eGFRcr was associated with lower odds of self-reported frailty in univariate and multivariable analysis (OR 0.80 per 10 ml/min/1.73 m^2^ (95 % CI 0.67–0.94) in multivariable analysis) (Table 2). When eGFRcr was modeled using categories, individuals with eGFRcr ≤ 29 ml/min/1.73 m^2^ were more likely to be frail by self-report (OR 3.20 95 % CI 1.61–6.40).

Associations of eGFRcys and covariates with self-reported frailty were slightly lower compared to those observed with both mGFR and eGFRcr. Higher eGFRcys was associated with lower odds of self-reported frailty in univariate and multivariable analysis (Table 2). When eGFRcys was modeled using categories, individuals with eGFRcys ≤ 29 ml/min/1.73 m^2^ were more likely to be frail by self-report (OR 2.30 95 % CI 1.11–4.70).

The association between GFR and frailty did not differ significantly according to age or sex for any method of assessing GFR (*p*-values for interactions >0.05). The potential for non-linearity of the association of GFR with frailty was examined using a quadratic term in the above models and by examining cubic splines, neither of which differed from the linear models presented. The concordance c statistic (area under the receiver operating characteristics [ROC] curve) for predicting frailty by self-report for the base model was 0. 66 (95 % CI, 0.60 to 0.71) (Fig. 2, Table 4). Adding mGFR (0.68, 95 % CI, 0.62 to 0.73), eGFRcr (0.67, 95 % CI 0.61 to 0.72) or eGFRcys (0.67, 95 % CI 0.62 to 0.72) to the model (as a categorical variables) did not significantly change the c statistic.

**Table 3:** Cox proportional hazard models of the association of self-reported frailty with death

Hazard ratio (95 % CI)				
Variables	Unadjusted for GFR	Adjusted for mGFR	Adjusted for eGFRcr	Adjusted for eGFRcys
Not frail (reference)	1.00	–	–	–
Intermediate frail	1.47 (1.14–1.90)	1.43 (1.11–1.83)	1.45 (1.13–1.87)	1.42 (1.10–1.83)
Frail	1.71 (1.26–2.30)	1.48 (1.08–2.00)	1.57 (1.15–2.10)	1.51 (1.10–2.10)

*Abbreviations*: *mGFR* measured glomerular filtration rate by Iothalamate, *eGFRcr* creatinine estimated glomerular filtration rate, eGFRcys by CKD-EPI equation. All models are adjusted for urine protein, sex, age, race, BMI; body mass index and albumin quartiles and group randomization by diet and blood pressure assignment. All GFR measures are per 10 ml/min/1.73 m2

**Table 4:** Area under the ROC curve (c statistic) for association of GFR with frailty models

ROC model	AUC	95 % Confidence limits	*P*-Value
Base model	0.66	0.60 to 0.71	–
Base model + mGFR	0.68	0.63 to 0.74	0.08
Base model + eGFRcr	0.68	0.63 to 0.73	0.08
Base model + eGFRcys	0.67	0.61 to 0.72	0.40

### Association of self-reported frailty with risk of death

Three hundred seventy-one (45 %) patients died before the end of 2007 (median follow-up 17 years (IQR 11–18)). Self-reported frailty was associated with a higher risk of death in multivariable modeling (HR 1.71 95 % CI 1.26–2.30) when kidney function was not included as a covariate (Table 3). Additionally adjusting for mGFR moderately attenuated the association of self-reported frailty with death (HR 1.48, CI 1.08–2.00). However, adjusting for either measure of eGFR yielded similar point estimates (HR 1.57, 95 % CI 1.15–2.10 for eGFRcr vs HR 1.51, 95 % CI 1.10–2.10 for eGFRcys). Intermediate self-reported frailty was also associated with a higher risk of death (HR1.47, 95 % CI 1.14–1.90 compared to non-frail participants) (Table 2), and adjustment for mGFR, eGFRcr, and eGFRcys yielded similar associations (Table 2).

## Discussion

We found a strong association between mGFR and self-reported frailty, such that patients with better kidney function were less likely to be frail by self-report even in healthy clinical trial study participants with relatively advanced CKD. Although similar when kidney function was modelled with mGFR or eGFR, the point estimate was strongest with mGFR. Regardless of which GFR variable was used as a covariate, the hazard ratios for the association of self-reported frailty with death were similar.

Our examination of associations using mGFR, eGFRcr and eGFRcys was novel. A common limitation of previous studies of frailty in the CKD population was the use of creatinine-based measures of kidney function [2, 5]. Because muscle mass is associated with serum creatinine and inversely with frailty, use of creatinine-based measures of kidney function may produce inaccurate associations. However, in this study we found similar associations using mGFR and eGFR. Our results suggest that the relationship between self-reported frailty and kidney function may not be particularly sensitive to method of measurement or estimation of GFR. Informally comparing the association of self-reported frailty with all three renal function measures suggests there was no difference between the three measures of kidney function. It is possible, however, that eGFRcr might not perform as well in a more heterogeneous group of patients with CKD as might be encountered in a community-based cohort [13] or in clinical practice and that eGFRcys may have non-GFR-related variability not observed in these analyses [14].

Direct comparisons of the prevalence of frailty across CKD populations are hampered by different characteristics of the cohorts (e.g., age range) and different definitions of frailty. Specifically, the prevalence of frailty is higher using definitions based on self-reported physical function compared to frailty defined by direct measures of physical performance [15]. Nevertheless, it is interesting that our estimate of frailty based on self-report is similar to that reported in the Seattle Kidney Study (SKS), which used an adapted fried criteria [1, 10]. We found a prevalence of frailty among MDRD participants of 16 % using a self-reported physical function based definition, whereas the frailty prevalence in the Seattle Kidney Study (SKS) was 14 % (95 % CI 10.5–18.2 %) based on direct measures of physical performance in a population with a larger comorbidity burden. Surprisingly, although MDRD clinical trial participants were healthier and younger than individuals in typical general elderly and ESRD cohorts, the prevalence of self-reported frailty among MDRD participants was double that of community elders [5, 10] and similar to that of other CKD cohorts with higher comorbidity burden [2]. As anticipated, the prevalence of self-reported frailty among MDRD participants was less than among ESRD cohorts, in which estimates range from 42 % to 73 % [3, 4, 15, 16].

The association of the severity of CKD with frailty has been previously evaluated using estimates of renal function. Similar to our findings with GFR modeled continuously, these studies have shown patients with more severe renal disease to have a higher likelihood of frailty [1, 13, 17]. To our knowledge, ours is the first study to test association of GFR with frailty based on three different methods for assessing renal function. Our study suggests associations of eGFRcys with frailty may have a similar magnitude of association using directly measured renal function. Thus studies that have used cystatin C as a measure of renal function may have provided reasonably calibrated findings to estimates using direct renal function measures [1, 13].

The expectation that frailty would be associated with a higher risk of death was based on a conceptual framework in which frailty is a marker of accelerated loss of functional reserve above and beyond what occurs as a result of kidney disease. Although other cohort studies have evaluated the association of frailty with risk of death among patients with CKD [1, 4, 16], the long period of follow-up in our study was unique, and our results show that frailty even by self-report is predictive of adverse outcomes even over longer term follow up. Self-reported frailty was associated with higher mortality independent of kidney function, raising the possibility that frailty operates through mechanisms not related to CKD such as inflammation, oxidative stress, or endothelial dysfunction related to CKD or its sequelae [18–21]. Further research into the role of these processes will be important to understand the pathophysiology of frailty in the CKD population.

A number of limitations of our study should be acknowledged. Our definition of frailty was based on patients’ self-reported physical function rather than direct measures of physical performance. We and others have shown that use of self-reported function identifies more individuals as frail, but such definitions have been associated with mortality as in this report [3–5, 8]. We assessed self-reported frailty at baseline and did not update frailty status during the long follow-up period, which may have biased our findings towards less of an association with death. We used underweight based on standard criteria in place of the weight loss criterion in our frailty definition, which likely underestimates the contribution of this component of frailty [15] MDRD participants were younger and healthier than the general CKD population. Although the relative health of the cohort helped to minimize potential confounding due to burden of comorbid disease, the finding that creatinine-based eGFRcr and mGFR were similarly associated with self-reported frailty must be considered with caution as greater differences might be observed in less healthy or older cohorts. For example, in the CHS, findings were much stronger with cystatin C than creatinine.

## Conclusions

In summary, we found that worse kidney function was associated with higher prevalence of self-reported frailty in a cohort with a smaller burden of comorbidities than is generally present among patients with advanced CKD. Furthermore, the inverse association between kidney function and self-reported frailty was similar when kidney function was measured or estimated. Self-reported frailty was associated with higher risk of death even after adjusting for kidney function. There is a need for longitudinal and interventional studies to determine whether intervening on frailty can improve outcomes among patients with CKD.

## Appendix

**Table 5:** Frailty phenotype and self-reported frailty definition

*Components of frailty*	*CHS* [10] *(fried criteria) frail*	*WHI* [8] *(woods criteria) self-report frail*	*MDRD self-report frail*
*Slowness/weakness*	*Timed 15 –foot walk (slowest 20 %);1 point. Grip strength (lowest 20 %) 1 point*	*Rand-36 Physical Function scale (score < 75) 2 points*	*MDRD Quality of Life Questionnaire; 2 points*
*Poor endurance/exhaustion*	*Two items from the Center for Epidemiologic Studies Depression Scale: Everything I do is an effort. I cannot get going. Answers >3-4 days/wk to either question were scored positive for frailty 1 point*	*Rand-36 Vitality Scale (score < 55) Over past 4 weeks: Did you feel worn out? Did you feel tired? Did you have a lot of energy? Did you feel full of pep? 1 point*	*MDRD Patient Symptom Questionnaire (monthly): In the past month how often have you felt lack of pep and energy? Tiring easily, weakness?, (scale never(0), mild(33), moderate(66), severe(100)) 1 point*
*Physical activity*	*Modified Minnesota Leisure Time activities: Self report of whether a person performed any if 18 activities in the prior week. Kcal of energy expended in a week on leisure time activity was calculated. Those in the bottom quintile were deemed positive for frailty. (men exerting < 383 kcal; women exerting <270 kcal/week were scored positive for frailty.) 1 point*	*Detailed activity questionnaire assessing frequency and duration of walking and mild, moderate, and strenuous activities. Kcal of weekly energy expenditure was calculated (metabolic equivalent task hours core = kcal/wk x kg), and those in the lowest quartile were scored positive for frailty. 1 point*	*MDRD Leisure Time Physical Activity Questionnaire components measuring number of times per week each individual completed walking, leisure time, vigorous and moderate activity. Converted to metabolic equivalents (kcal/week x kg) those in the lowest quartile will be scored positive for frailty. 1 point*
*Unintentional weight loss*	*Self-reported weight loss > 10 pounds in previous year at baseline and measured weight loss > 4 % of body weight in prior year. 1 point*	*No measure was available at baseline. At follow-up, measured weight loss at Year 3 visit > 5 % and “yes” to question, “In the past two years, did you lose five or more pounds not on purpose at any time?” 1 point*	*No measure was available at baseline. At follow-up, measured weight loss at Year 1 visit > 5 % of standard body weight from B3 visit to a weight of 75-95 % of standard body weight and Question 9c/d from monthly exam related to weight change.1 point*
	*5 points*	*5 points*	*5 points*
	*≥3 points = Frail*	*≥3 points = Frail*	*≥3 points = Frail*
	*1*–*2 points = intermediate frail*	*1*–*2 points = intermediate frail*	*1*–*2 points = intermediate frail*
